# OsSLI1, a Homeodomain Containing Transcription Activator, Involves Abscisic Acid Related Stress Response in Rice (*Oryza sativa* L.)

**DOI:** 10.1155/2014/809353

**Published:** 2014-06-25

**Authors:** Xi Huang, Min Duan, Jiakai Liao, Xi Yuan, Hui Chen, Jiejie Feng, Ji Huang, Hong-Sheng Zhang

**Affiliations:** State Key Laboratory of Crop Genetics and Germplasm Enhancement, Nanjing Agricultural University, Nanjing 210095, China

## Abstract

Homeodomain-leucine zipper type I (HD-Zip I) proteins are involved in the regulation of plant development and response to environmental stresses. In this study, *OsSLI1* (*Oryza sativa stress largely induced 1*), encoding a member of the HD-Zip I subfamily, was isolated from rice. The expression of *OsSLI1* was dramatically induced by multiple abiotic stresses and exogenous abscisic acid (ABA). *In silico* sequence analysis discovered several *cis*-acting elements including multiple ABREs (ABA-responsive element binding factors) in the upstream promoter region of *OsSLI1*. The OsSLI1-GFP fusion protein was localized in the nucleus of rice protoplast cells and the transcriptional activity of OsSLI1 was confirmed by the yeast hybrid system. Further, it was found that *OsSLI1* expression was enhanced in an *ABI5-Like1* (*ABL1*) deficiency rice mutant *abl1* under stress conditions, suggesting that ABL1 probably negatively regulates *OsSLI1* gene expression. Moreover, it was found that *OsSLI1* was regulated in panicle development. Taken together, *OsSLI1* may be a transcriptional activator regulating stress-responsive gene expression and panicle development in rice.

## 1. Introduction

Transcription factors can bind to specific* cis*-elements in gene promoter regions. Through the activation or inhibition effects on gene expression, they play important roles in regulation of plant growth and development and morphogenesis, as well as responses to environment stimuli [1]. To date, the database of rice transcription factors (DRTF) contains 2,025 putative transcription factors (TF) in* Oryza sativa* L. ssp.* indica* and 2,384 in* Oryza sativa* L. ssp.* japonica*, distributed in 63 families [2]. The main transcription factor families among them (over 100 family members in* indica/japonica*) are AP2/EREBP (174/182), bHLH (157/184), bZIP (88/109), C2H2 (94/113), HB (84/103), MYB (136/138), NAC (157/149), and WRKY (111/113).

The Homeodomain-leucine zipper (HD-Zip) genes have diverse functions in plant development and have often been implicated in stress adaptation [3]. The HD-Zip genes are the most abundant group of homeobox (HB) genes in plants and do not exist in other eukaryotes [4]. A typical DNA binding domain of homeobox proteins contains 60 amino acids, known as homeodomain. The homeodomain folds into a characteristic 3D structure containing three alpha-helices, of which the second and third forma helix-turn-helix motif recognize and interact with specific DNA sequences as homodimers [5]. Agalou et al. identified 33 HD-Zip genes in rice and classified them into family I (14 genes), family II (14 genes), and family III (5 genes) [6]. The HD-Zip IV family in other higher plants has been previously described [7, 8].

Söderman et al. first identified an HD protein ATHB-7 from* Arabidopsis thaliana*, which was highly induced by salt, osmotic stress, and ABA treatment [9]. Later, Leeand Chun identified Athb-12, which shares over 80% identity with ATHB-7 in their homeodomain and Zip motifs [10]. Expression of* Athb-12* was induced by water stress and exogenous abscisic acid (ABA). Another HD-Zip gene,* ATHB6, *was expressed constitutively in seedlings but significantly upregulated in seedlings subjected to water deficit, osmotic stress, or exogenous treatment with ABA [11]. Athb-12 was also isolated by functional complementation of the NaCl-sensitive phenotype of a calcineurin (CaN-) deficient yeast mutant and it could increase NaCl tolerance but not osmotic stress tolerance of these mutant cells if expressed [12]. Zhu et al. isolated an* Arabidopsis mutant hos9-1* and suggested that HOS9 mediates cold tolerance through a CBF-independent pathway [13].

Beside the model plant* Arabidopsis*, a number of HD-Zip proteins involved in abiotic stress response were also identified in the last decade from diverse crop plants. An HD-Zip protein was identified closely related to low temperature stress in hot pepper (*Capsicum annuum L.*) [14]. HAHB4 belongs to the sunflower HD-Zip proteins subfamily I and is involved in drought response and ethylene-mediated senescence [15]. In cotton, the GhHB1 was isolated from a cotton root cDNA library. The expression level of the GhHB1 gene was dramatically increased in roots under treatment with 1% NaCl and exogenous abscisic acid [16]. Expression of* Nicotiana attenuata *NaHD20 was induced by multiple stress-associated stimuli including drought and wounding. It was predicted to be involved in leaf ABA accumulation during water stress, benzylacetone emission from flowers, and the timing of bolting and flower transitions [17]. Several drought-related members of the HD-Zip gene family were also characterized [6].

Rice is one of the most important food crops in the world, feeding half of the world's population. Salt and drought are two major abiotic stresses for agricultural production [18]. With a relatively small genome size, rice is an ideal model plant for molecular and genetic research. It is critical to identify the roles of stress-induced genes in order to develop transgenic crops that have enhanced tolerance to unfavorable growth conditions or help breeding programs by molecular assistant selection [19].

In the present study,* OsSLI1* encoding a member of HD-Zip I subfamily was analyzed. Expression of* OsSLI1* was regulated by panicle development and dramatically induced by salt, drought, and ABA treatment. An ABI5-like transcription factor ABL1 probably negatively regulates* OsSLI1* gene expression in rice under stress conditions.* OsSLI1 *may be a transcriptional activator regulating stress-responsive gene expression and panicle development in rice.

## 2. Materials and Methods

### 2.1. Plant Materials and Stress Treatments

The seeds of rice (*Oryza sativa* L. sub.* japonica*) cultivar Jiucaiqing, the rice ABI5-Like1 (ABL1) deficiency mutant,* abl1*, and its wild type Zhonghua 11 (ZH11,* japonica*) were sterilized in 0.1% HgCl_2_ and germinated at 30°C. The seedlings were cultured with Yoshida's culture solution in growth chamber as previously described [20]. The 2-week-old seedlings were used for subsequent stress treatments. For H_2_O_2_, salt, PEG, and ABA treatments, seedlings were transferred to nutrients solutions containing 100 mM H_2_O_2_, 100 mM NaCl, 20% (w/v) PEG6000, and 50 *μ*M ABA, respectively. For cold and heat treatments, seedlings were transferred to the growth chamber with the temperature of 4°C and 42°C, respectively. For drought treatment, germinated seeds were planted in 15 cm diameter pots filled with soil, watered daily for 2 weeks; watering was then withheld to simulate a natural dehydration environment. All samples were collected at various times after treatments, immediately freezed in liquid nitrogen, and stored in −80°C freezer.

### 2.2. RNA Isolation and First Strand cDNA Synthesis

Total RNA was extracted from various stress-treated rice seedlings using Trizol reagent (Invitrogen, USA) according to the manufacturer's protocol. The RNA was subsequently treated with DNase I (Promega, USA) to remove the remaining genomic DNA. The first strand cDNA was synthesized with 2 *μ*g total RNA using the reverse transcription system (Promega, USA).

### 2.3. Cloning of OsSLI1

Pairs of primers (forward: 5′-TAGTGCTCTGGTCTTACATT-3′ and reverse: 5′-TACGCTGATTGATTAGCATG-3′) were employed to clone* OsSLI1* by RT-PCR approach. The PCR conditions for amplifying* OsSLI1* were as follows: 5 min predenaturation at 95°C, 32 cycles of 20 s at 95°C, 20 s at 56°C, and 50 s at 72°C, and a final extension for 5 min at 72°C. The PCR product was purified and cloned into pMD19-T vector (Takara, Japan) for sequencing.

### 2.4. Quantitative RT-PCR Assay

qRT-PCRs were carried out on an Applied Biosystems 7500 Fast Real Time PCR System (Applied Biosystems). The qRT-PCR mixture of 20 *μ*L was prepared using the FastStart Universal SYBR Green Master (Rox) (Roche Diagnostics). Quantification of gene expression was done using the comparative CT method. Experiments were performed in triplicate and the results were represented by means ± SE of three replicates. 18S rRNA was used as an internal control [21]. The primers of 18S rRNA were as follows: forward primer: 5′-ATGGTGGTGACGGGTGAC-3′; reverse primer: 5′-CAGACACTAAAGCGCCCGGTA-3′.

### 2.5. Multiple Sequence Alignment and Phylogenetic Analysis

Alignment of rice or other plant homeodomain protein sequences were performed with ClustalX program (ver 2.1). The phylogenetic tree was constructed with MEGA program (ver 4.0) by neighbor-joining (NJ) method. The parameters pairwise deletion and p-distance model were used. Bootstrap test of phylogeny was performed with 1,000 replicates.

### 2.6. Subcellular Localization

A green florescent protein (GFP) fusion protein was constructed using the full-length* OsSLI1 *cDNA clone with a C-terminal fusion of the GFP clone under the control of CaMV 35S promoter. Rice protoplast preparation and transformation were performed as previously described [22]. The subcellular distribution of the GFP fusion protein was examined by confocal laser scanning microscopy (Leica, TCS SP2). Cells were labeled with the DNA dye 4,6-diamidino-2-phenylindole (DAPI) to visualize the nucleus.

### 2.7. Transcriptional Activation Assay

The transcriptional activation activity of OsSLI1 was determined as previously described [23]. The full-length ORF of* OsSLI1* was cloned into the pGBKT7 vector containing the GAL4 DNA binding domain to obtain pGBKT7-OsSLI1. According to the protocol of the manufacturer (Stratagene, USA), pGBKT7-OsSLI1 and the negative control pGBKT7 vector were transferred into the yeast strain AH109, respectively. The transformed yeast cells were grown on SD glucose medium plates lacking Trp. Single colony (2 mm diameter) was picked and incubated in SD glucose liquid medium lacking Trp at 30°C for 2-3 days. Proper volume of the culture was transferred on SD glucose medium plate with 5 *μ*M 3-AT and lacking Ade and His and incubated at 30°C for 2-3 days. The transactivation activity of each protein was evaluated according to its growth status and *β*-galactosidase assay (Yeast Protocols Handbook, Clontech).

### 2.8. *In Silico* Sequence Analysis

For promoter sequence analysis, the 2,000-bp upstream sequence of* OsSLI1 *was analyzed by Matinspector program at Genomatix website (http://www.genomatix.de/). The coexpression analysis was performed both with Rice Oligo Array Database (http://ricearray.org/) based on 1,081 rice Affymetrix microarray data (NCBI GEO AC: GPL2025) and by genevestigator (https://www.genevestigator.com/gv/). The coexpressed genes with* OsSLI1 *were selected with a Pearson's correlation coefficient (PCC) cutoff of 0.6 in both results. The expression changes of coexpressed genes under cold, salt, and drought stresses were investigated using GEO (Gene Expression Omnibus) datasets. For promoter analysis, the 1,000-bp upstream sequences of each coexpressed gene were analyzed by our in-home program.

## 3. Results and Discussion

### 3.1. OsSLI1 Belongs to HD-Zip Family

Through a microarray approach (data not shown), an EST probe (ID: Os.49245.1.S1_at) was found with high induction levels in two-week-old rice seedlings by 100 mM NaCl (227-fold induction), 50 *μ*M ABA (174-fold induction), and 20% (w/v) PEG6000 (134.5-fold induction). This EST was chosen for further research due to its dramatic expression increase under abiotic stress treatments. Database searching showed that this EST sequence encodes a putative homeodomain containing protein. Here, we named this gene* OsSLI1 stress largely induced 1* as its expression is largely induced by multiple abiotic stresses and ABA treatment.

To elucidate the function of* OsSLI1*, the full-length* OsSLI1* cDNA was amplified from rice by RT-PCR.* OsSLI1 *is located on chromosome 2. The 783 bp ORF was predicted to produce a 261 amino acids protein with the calculated molecular mass of 28.54 kDa and the PI of 4.61. Putative conserved domains were detected through BLAST program of NCBI, including a 56-AA HD domain (65–121) for DNA binding and a 43-AA Zip domain (122–164) for protein-protein interactions. Previously, Agalou et al. investigated HD-Zip gene family in rice, confirmed 31 expressed genes of the total 33 family members, and assigned 14 of them, including* OsSLI1 *into HD-Zip I subfamily [6].

**Figure 1:**
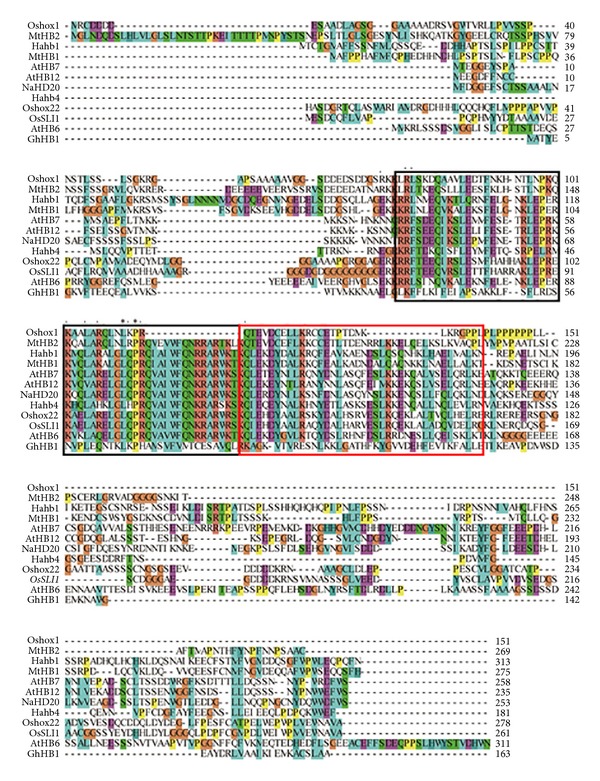
Sequence alignment of stress-related HD-Zip proteins in plants. The HD domain and Zip domains are boxed in black and red, respectively. GenBank accession numbers for the sequences: AtHB6 (AT2G22430), AtHB7 (AT2G46680), AtHB12 (AT3G61890), GhHB1 (EF151309), Hahb1 (AAA63765), Hahb4 (AAA63768), MtHB1 (ABO47743), MtHB2 (ACJ84212), NaHD20 (HM107874), Oshox1 (AAG13598), Oshox22 (AAO72559), and OsSLI1 (NP_001047582).

**Figure 2:**
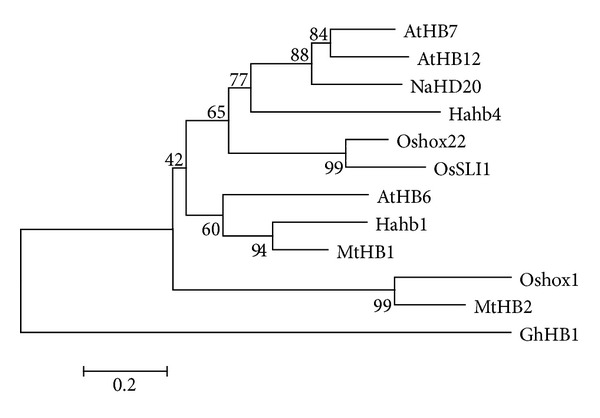
Phylogenetic analysis of stress-related HD-Zip proteins in plants. The neighbor-joining tree was constructed with MEGA 4.0. Branch numbers represent a percentage of the bootstrap values in 1,000 sampling replicates and the scale bar indicates branch length.

Multiple stress-related HD-Zip protein sequences were collected to perform multiple sequence alignment and phylogenetic analysis (Figures 1 and 2). Sequence alignment showed that HD domain and Zip domain are highly conserved among all the 12 proteins. The phylogenetic analysis revealed that OsSLI1 was highly close to MtHB2 and Oshox22. Previous study suggests that MtHB2, which were identified from* Medicago truncatula*, may play a negative role in regulation of abiotic stress response in* Arabidopsis* [24]. However, Oshox22 affects ABA biosynthesis and regulates drought and salt responses through ABA-mediated signal transduction pathways with a positive role [25]. These results indicate that HD-ZIP proteins may play different roles in stress responses in plants.

### 3.2. Expression of OsSLI1 in Different Rice Tissues

**Figure 3:**
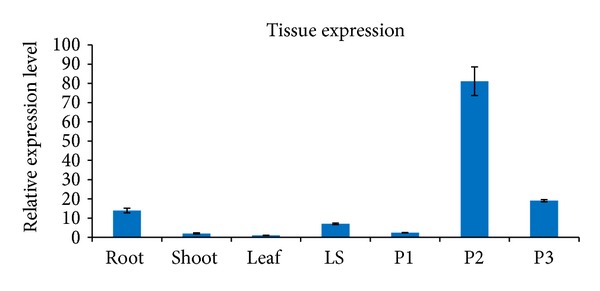
Expression patterns of* OsSLI1* in various rice tissues. P1: 3 cm panicles, P2: 8 cm panicles, and P3: 12 cm panicles.

For tissue specific expression analysis, root and shoot tissues from two-week-old seedlings were collected. Booting stage leaves (Leaf), leave sheath (LS), and panicles with the lengths of 3 cm (P1), 8 cm (P2), and 12 cm (P3) were also collected from unstressed field grown plants. Quantitative RT-PCR result showed that, in the seedlings,* OsSLI1* expression was mainly in root. The root tissue accumulates 7 times of* OsSLI1* transcripts than in shoot tissue. In panicles, the expression changes in different development stages, from relative low in 3 cm young panicles to a 33-fold increase in 8 cm panicles, and the 12 cm panicles still accumulate 8-fold* OsSLI1* transcripts compared to 3 cm young panicles tissue (Figure 3).

It is interesting that* OsSLI1* transcript accumulates much more in reproduction organs compared to other tissues in rice. In* Arabidopsis, *transgenic lines overexpressing sunflower* HAHB4* gene, a compact inflorescence, and short pedicels were observed [26]. Under control growth conditions, NaHD20-silenced plants presented a delay in bolting compared with empty vector control plants. The production of the first opened buds was delayed in NaHD20-silenced plants; the number was also significantly reduced [17]. Considering that HAHB4, NaHD20, and OsSLI1 were all classified into HD-Zip subfamily I, it is of interest to investigate whether members of this subfamily also are involved in reproduction organ development in plants.

### 3.3. Expression of OsSLI1 under Different Abiotic Stress Conditions

Since microarray data showed that* OsSLI1* was largely induced by drought and salt stresses and ABA treatment, we investigated the expression pattern of the gene in response to various abiotic stresses. The rice cultivar Jiucaiqing, which was tolerant to drought and salt stresses [27, 28], was used in abiotic stress treatment.

**Figure 4:**
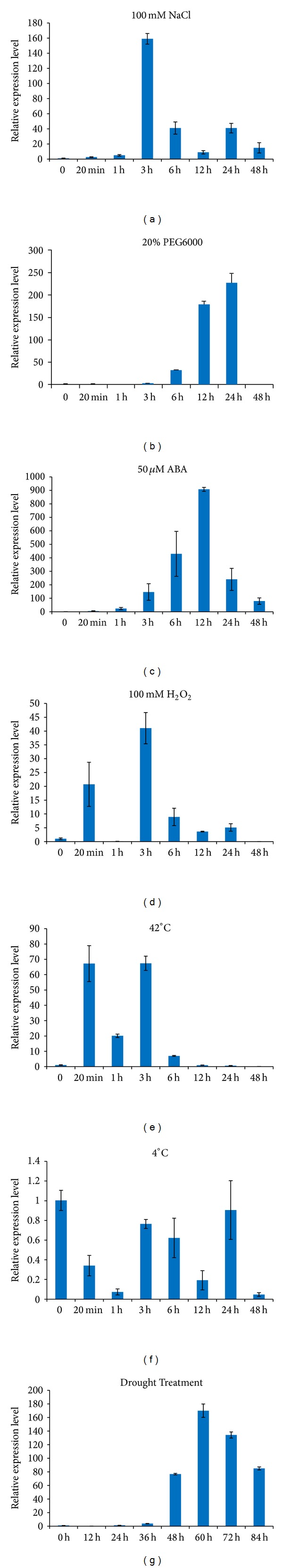
Expression change of* OsSLI1* in rice seedlingsunder different treatments. (a) salt stress (100 mM NaCl); (b) osmotic stress (20% PEG6000); (c) ABA treatment (50 *μ*M ABA); (d) oxidative stress (100 mM H_2_O_2_); (e) high temperature (42°C); (f) low temperature(4°C); and (g) drought treatment.

Quantitative RT-PCR assay showed that* OsSLI1* expression was highly induced by salt, PEG, H_2_O_2_, high temperature, and ABA treatment but not by cold treatment. For salt treatment,* OsSLI1* reached a 159-fold expression peak after a 3-hour treatment and maintained high expression till 48 hours. For PEG treatment,* OsSLI1* transcript was slowly accumulated, starting responsiveness to the treatment after 6 hours, and reached the peak at 24 hours.* OsSLI1* expression profiles under H_2_O_2_ and high temperature 11 treatment were very similar, showing a two-peak induction pattern at 20 min and a 3-hour treatment with an expression decrease in 1-hour treatment. 50 *μ*M exogenous ABA was applied to the seedlings as a signal molecule.* OsSLI1* expression was induced by 907-fold after 12 hours of induction and then decreased to 78-fold till 48 hours. For low temperature treatment,* OsSLI1* expression changes were not dramatic compared to other stresses (Figure 4). For drought,* OsSLI1* expression was induced 4 times at 36 hours as compare to unstressed samples and reached its peak of 170-fold induction at 60 hours; then the expression decreased slowly and still maintained an 85-fold expression level at 84 hours.

### 3.4. *cis*-Acting Element Analysis

**Figure 5:**
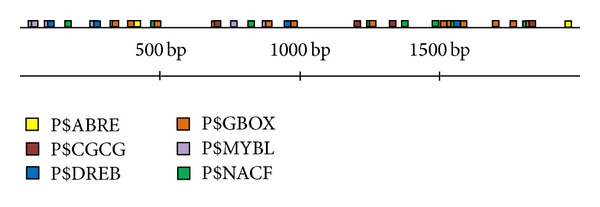
Distribution of stress-related* cis*-acting elements in the 2,000 bp promoter region of* OsSLI1. *Different* cis*-elements for TFs were labeled with different colors. Right end 2,000 bp is the transcriptional start site (TSS) of* OsSLI1.*

The upstream 2,000 bp DNA sequence of* OsSLI1* was used for* cis*-acting element analysis. Some putative stress-related* cis*-elements, such as ABRE (ABA-responsive element binding factors), CGCG (calmodulin binding/CGCG box binding proteins), CRT/DREB (dehydration responsive element binding factors), GBOX (plant G-box/C-box bZIP proteins), MYBL (MYB-like proteins), and NACF (plant specific NAC transcription factors), were found (Figure 5). A total of 15 ABREs were found in this region 13 of which are located within the upstream 1,000 bp, indicating that the expression of* OsSLI1* might be tightly regulated by ABF/bZIP type transcription factors. These stress-related* cis*-elements may be responsive for stress-responsive expression of* OsSLI1*.

### 3.5. Subcellular Localization and Transcriptional Activation Analysis

**Figure 6:**
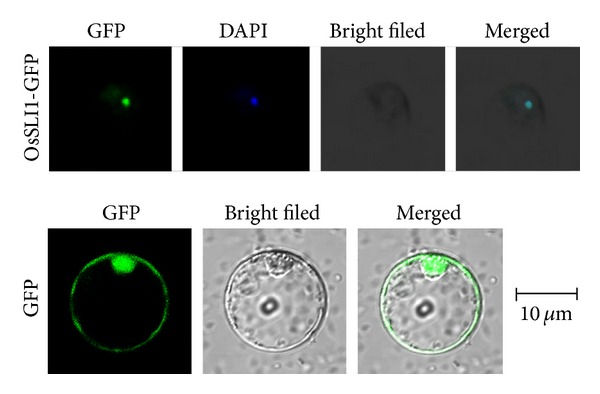
Nuclear localization of OsSLI1-GFP fusion protein. Constructs of 35S:OsSLI1-GFP (above) and 35S:GFP were transformed into rice protoplast cells, respectively. The GFP and DAPI signals were observed by confocal microscopy.

To control downstream gene expression, most transcription factors locates in the nucleus. A* pCaMV 35S : OsSLI1-GFP* plasmid was constructed and introduced into rice protoplasts through PEG mediated transient transformation. The fusion protein was detected in rice protoplast nucleus part, which was indicated by DAPI staining. This observation suggests that OsSLI1 is a nuclear-localized protein, in consistency with its potential function as a DNA binding transcription factor (Figure 6).

**Figure 7:**
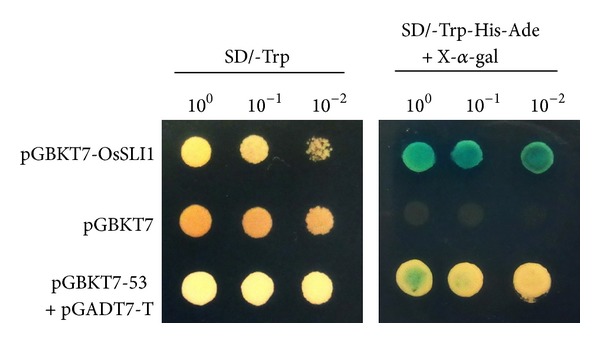
Transcriptional activation analysis of OsSLI1 in yeast. The recombinant vector pGBKT7-OsSLI1 was transferred into yeast strain AH109. Transcriptional activation ability of OsSLI1 was analyzed by grown on SD/-Trp and SD/-Trp-His-Ade/5 mM 3-AT plates. Negative control: yeast strain AH109 with pGBKT7 vector; positive control: yeast strain AH109 with pGBKT7-53 and pGADT7-T vectors.

Yeast hybrid system was employed to investigate the transcriptional activity of OsSLI1. As shown in Figure 7, all transformants grew well on SD/Trp medium. However, only transformants containing pGBKT7-OsSLI1 could grow on SD/Trp-/His-/5 mM 3-AT medium, whereas the yeast cells transformed with pGBDT7 control failed to grow. These results were consistent with the *β*-galactosidase activity analysis, and indicated that OsSLI1 had transactivation activity.

### 3.6. Expression of OsSLI1 in Rice abl1 Mutant under Abiotic Stress

**Figure 8:**
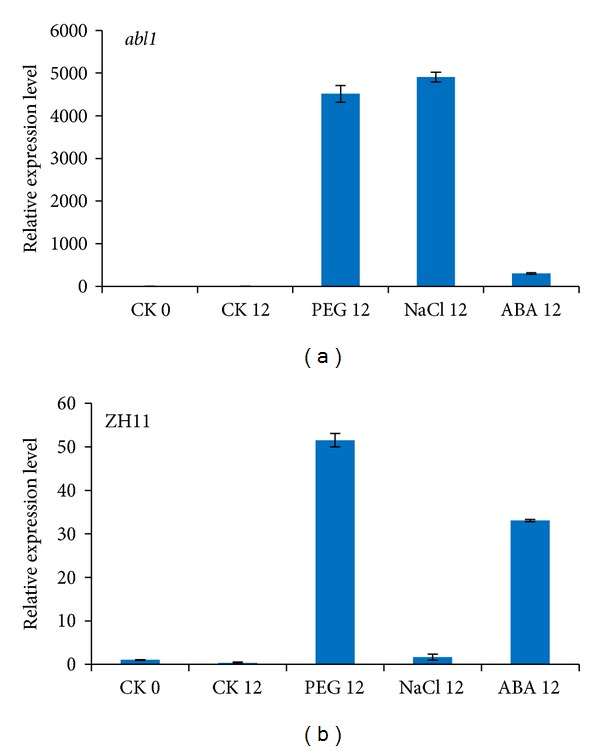
Expression change of* OsSLI1* in rice* abl1* mutant (a) and WT ZH11 (b) seedlings under different treatments.

The rice ABI5-Like1 (ABL1) deficiency mutant,* abl1*, shows suppressed ABA responses [29]. Since evidences from previous data suggest* OsSLI1's *expression pattern is ABA related, it is interesting to analyze whether* OsSLI1* is involved in ABL1 signaling. We analyzed* OsSLI1 *gene expression in* abl1* and wild-type rice ZH11 under salt and drought stresses. After 12 hour of induction by PEG,* OsSLI1* gene expression increased to 4,515-folds compared to untreated samples in* abl1*, while in WT its expression only increased 51 times. For NaCl treatment, 12 hours of induction in* abl1 *showed 4,900 times higher than untreated samples while, in WT, it was only induced 1.6 times. Similarly,* OsSLI1* expression was 302- and 33-fold increased by ABA treatment in* abl1 *and WT, respectively.* OsSLI1 *expression was enhanced in* abl1* mutant under both normal and stress conditions (Figure 8), suggesting that ABL1 probably negatively regulates* OsSLI1* gene expression.

### 3.7. Coexpression Analysis

A total of 96 positively coexpressed genes and one negatively coexpressed gene were selected with Pearson's correlation coefficient higher than 0.6 from the* OsSLI1 *coexpression gene data by rice array database. Coexpression analysis by Genevestigator discovered 35 genes positively coexpressed with* OsSLI1*. Six genes were found in both results (Table 1). Five of the six genes showed upregulated response to all three abiotic stresses. Only LOC_Os06g41360, a phosphoribosyltransferase, was slightly induced by drought and salt treatments and inhibited by cold stress. For the other five genes, one gene (LOC_Os07g05940) encodes NCED, a key enzyme in ABA biosynthesis pathway showed upregulation by cold. The top 2 genes with a higher PCC value encode protein phosphatase 2C, which negatively regulate ABA signaling at an early step in the pathway [30]. The result of coexpression analysis suggests the potential function of* OsSLI1* in ABA related stress response in rice.

To investigate whether those coexpressed genes are the targets of OsSL1, we analyzed the existences of putative HD-ZIP binding sites (CAATNATTG) in promoter regions of 96 coexpressed genes. We only found that one gene (LOC_Os02g54254) may contain the putative HD-ZIP binding site in its promoter region. Therefore, most of the coexpressed genes may not be the direct targets of OsSL1.

## 4. Conclusion

The plant-specific homeodomain-leucine zipper type I (HD-Zip I) proteins play important roles in plant development and stress responses. In this research,* OsSLI1* encoding an HD-ZIP I protein was isolated from rice. Time-course expression analysis indicated that* OsSLI1 *expression was induced very quickly and dramatically by various abiotic stresses including salt, drought, osmotic treatment, and exogenous abscisic acid. The promoter sequence of* OsSLI1* harbors several* cis*-elements including high density of ABA-responsive elements, but the well-known CRT/DREB element is much less. Further, it was found that* OsSLI1 *expression was enhanced in an ABI5-Like1 (ABL1) deficiency mutant* abl1* under both normal and stress conditions, suggesting that ABL1 probably negatively regulates* OsSLI1* gene expression. It was found that* OsSLI1* was also regulated in panicle development. The OsSLI1-GFP fusion protein was localized in the nucleus of rice protoplast cell. Yeast hybrid assays revealed that OsSLI1 had transcription activation activity, indicating that OsSLI1 functions as a transcription activator. Taken together,* OsSLI1 *may be a transcriptional activator regulating stress-responsive gene expression and panicle development in rice. Further studies with* OsSLI1* transgenic plants will be necessary to elucidate the roles of OsSLI1 in stress responses and panicle development in rice.

## Figures and Tables

**Table 1:** List of genes coexpressed with *OsSLI1*.

Gene ID	Annotation	PCC^1^	PCC^2^	Drought	Salt	Cold
LOC_Os09g15670	Protein phosphatase 2C	0.7719	0.64	7.16	6.29	4.27
LOC_Os03g16170	Protein phosphatase 2C	0.6799	0.68	5.69	4.44	3.64
LOC_Os07g05940	9-*cis*-Epoxycarotenoid dioxygenase, chloroplast precursor	0.6140	0.70	6.92	5.93	6.45
LOC_Os07g48830	Glycosyltransferase 8 domain containing protein	0.6805	0.63	5.07	3.97	3.78
LOC_Os06g41360	Phosphoribosyltransferase	0.6511	0.62	1.98	1.21	−0.42
LOC_Os05g41490	Circadian clock coupling factor ZGT	0.6115	0.61	6.45	5.88	3.01

^1,2^PCC: Pearson's correlation coefficient. 1: rice array; 2: genevestigator.

The data in the table indicate the log⁡2-fold of the microarray data in rice seedlings under drought, salt, or cold stress relative to that under control condition. The normalized microarray data were obtained from GEO database under accession number GSE6901.
